# Management of Severe Leptospirosis in the Intensive Care Unit: A Case Report and Review of Diagnostic and Therapeutic Challenges

**DOI:** 10.7759/cureus.84140

**Published:** 2025-05-14

**Authors:** João Trepa, André B Ribeiro, Bárbara A Quental, Miguel Sequeira, Ana Albuquerque

**Affiliations:** 1 Intensive Care Unit, Unidade Local de Saúde de Viseu Dão-Lafões, Viseu, PRT; 2 Department of Emergency Medicine, Unidade Local de Saúde de Viseu Dão-Lafões, Viseu, PRT

**Keywords:** acute respiratory distress syndrome (ards), alveolar hemorrhage, intensive care, leptospirosis, sepsis

## Abstract

Severe leptospirosis is a zoonotic disease with a global distribution, which can be severe, requiring intensive care management due to its potential for multi-organ dysfunction. This case report describes a 57-year-old male patient with a history of alcohol abuse, dyslipidemia, and hypertension, who presented with severe leptospirosis. The patient exhibited symptoms including generalized myalgia, abdominal pain, vomiting, and jaundice. Initial laboratory results revealed significant abnormalities, such as elevated liver enzymes, high bilirubin levels, and severe thrombocytopenia. Despite broad-spectrum antimicrobial therapy, the patient developed acute respiratory distress syndrome and required admission to the intensive care unit. Organ support interventions included mechanical ventilation with protective strategies, prone positioning, and continuous renal replacement therapy. The patient's condition was further complicated by an anuric state, delaying confirmation of leptospirosis through polymerase chain reaction (PCR) testing. Empiric treatment with ceftriaxone and corticosteroids was initiated, leading to gradual clinical improvement. The patient was extubated after 13 days but developed severe critical illness-related myopathy, which led to prolonged hospital admission, but eventually made a full recovery. This case highlights the importance of timely diagnosis and intervention in severe leptospirosis, emphasizing the role of intensive care management in improving patient outcomes. Further research is needed to establish standardized treatment protocols for severe leptospirosis, particularly concerning antimicrobials and corticosteroids.

## Introduction

Leptospirosis is a zoonosis with a worldwide distribution and a variety of maintenance hosts, the most common being small rodents. It is most prevalent in tropical and subtropical regions because a warm and humid environment favours its survival. *Leptospires* can enter the body in several ways, most commonly by swallowing contaminated water, but also through cuts and abrasions, mucous membranes, or conjunctivae, and inhalation of aerosols. Invasion across the epithelium is followed by proliferation and dissemination, including the central nervous system. Meningitis and encephalitis are common, but leptospirosis is rarely on the differential diagnosis [1].

The mechanism by which *Leptospires *cause disease is poorly understood. Virulence factors differ among species and serovars, contributing to the variability in disease severity. Advancements in molecular biology have facilitated the classification of the genus *Leptospira*. Currently, there are 68 named species within this genus, encompassing both pathogenic and non-pathogenic strains, and over 300 serovars [2]. The most common species responsible for human leptospirosis are *L. interrogans* and *L. borgpetersenii*. In addition to bacteria-related mechanisms of injury, host susceptibility and variability factors also influence immune and autoimmune mechanisms, which in turn shape clinical manifestations [3].

According to the European Center for Disease Prevention and Control, the incidence of leptospirosis in Europe in 2022 was 0.18 confirmed cases per 100,000 population, with a case fatality rate of 0.7%. Portugal reports one of the highest incidence rates in Europe [4]. The disease is under-reported even in developed countries, one of the reasons being the wide spectrum of illness, ranging from asymptomatic (most cases) to a severe multisystem disease with a high mortality rate.

Severe disease can manifest in various forms, notably Weil’s disease, characterized by hepatic and renal dysfunction. One of the most fatal complications is severe pulmonary hemorrhagic syndrome (SPHS), with mortality rates ranging from 50% to 70% [5,6]. In SPHS, capillary endothelial damage leads to interstitial and intra-alveolar hemorrhage, causing diffuse alveolar damage and acute lung injury that can progress to respiratory distress syndrome (ARDS) and septic shock [7].

## Case presentation

**Figure 1:**
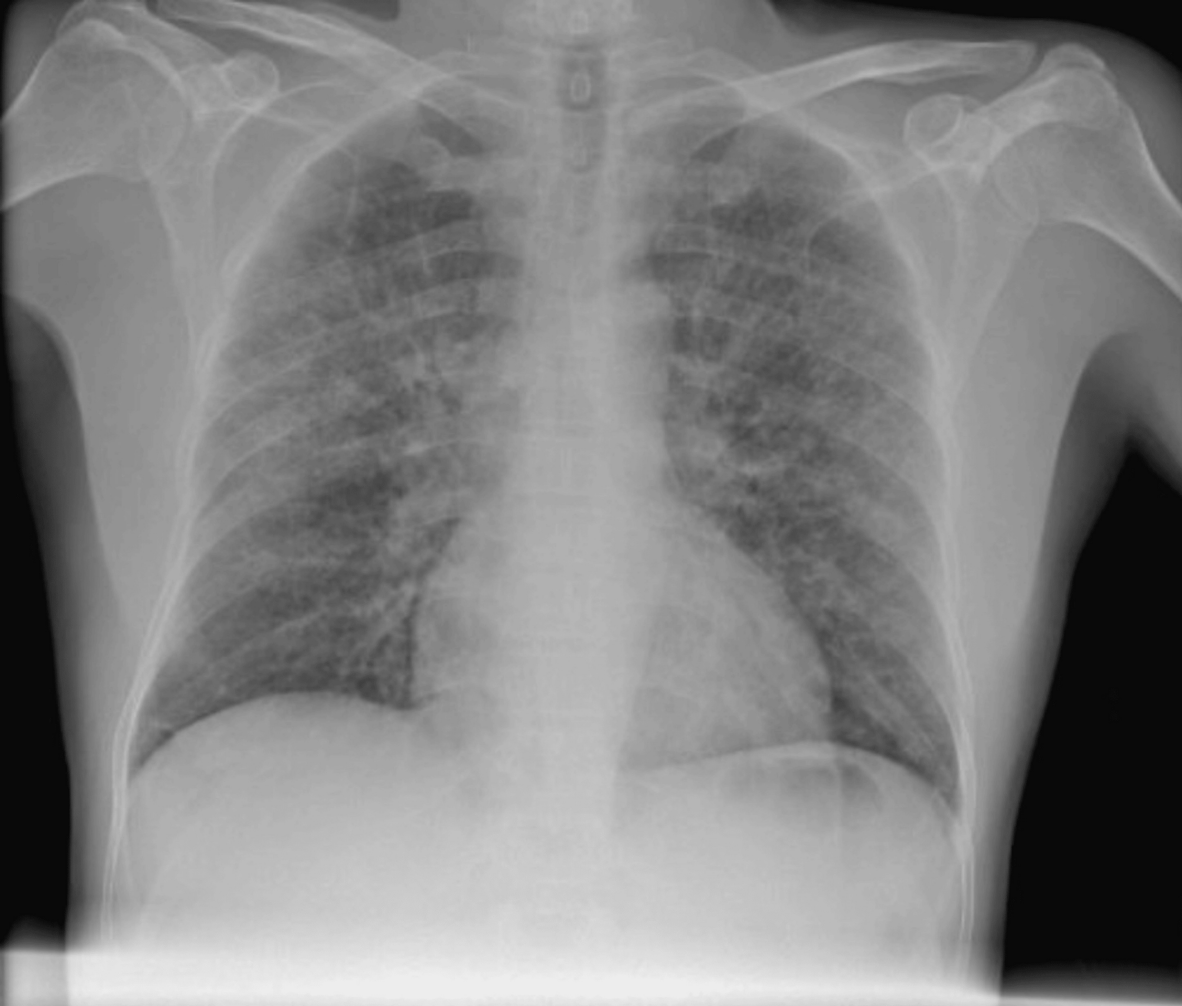
Chest radiograph of the patient in the Emergency Department Chest radiograph showing a diffuse infiltrative opacification pattern

**Figure 2:**
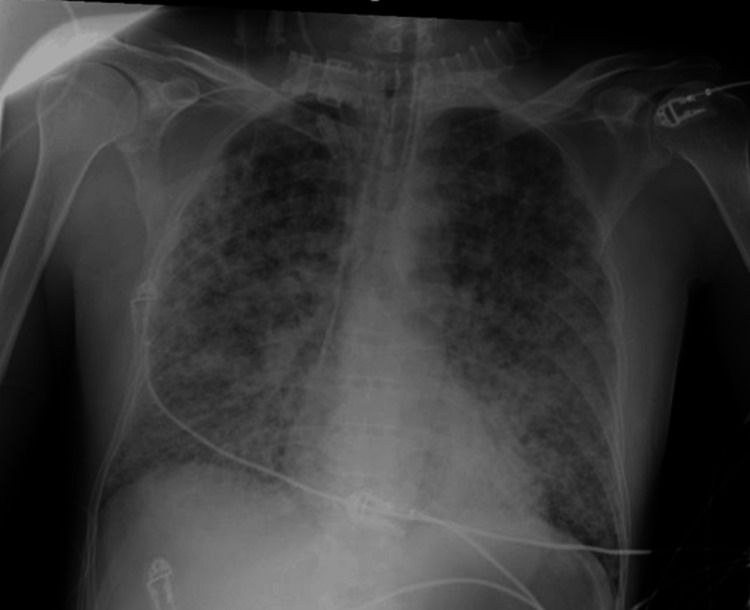
Chest radiograph taken five hours after intensive care unit admission Chest radiograph showing worsening diffuse infiltrative opacities with an endotracheal tube correctly positioned

**Figure 3:**
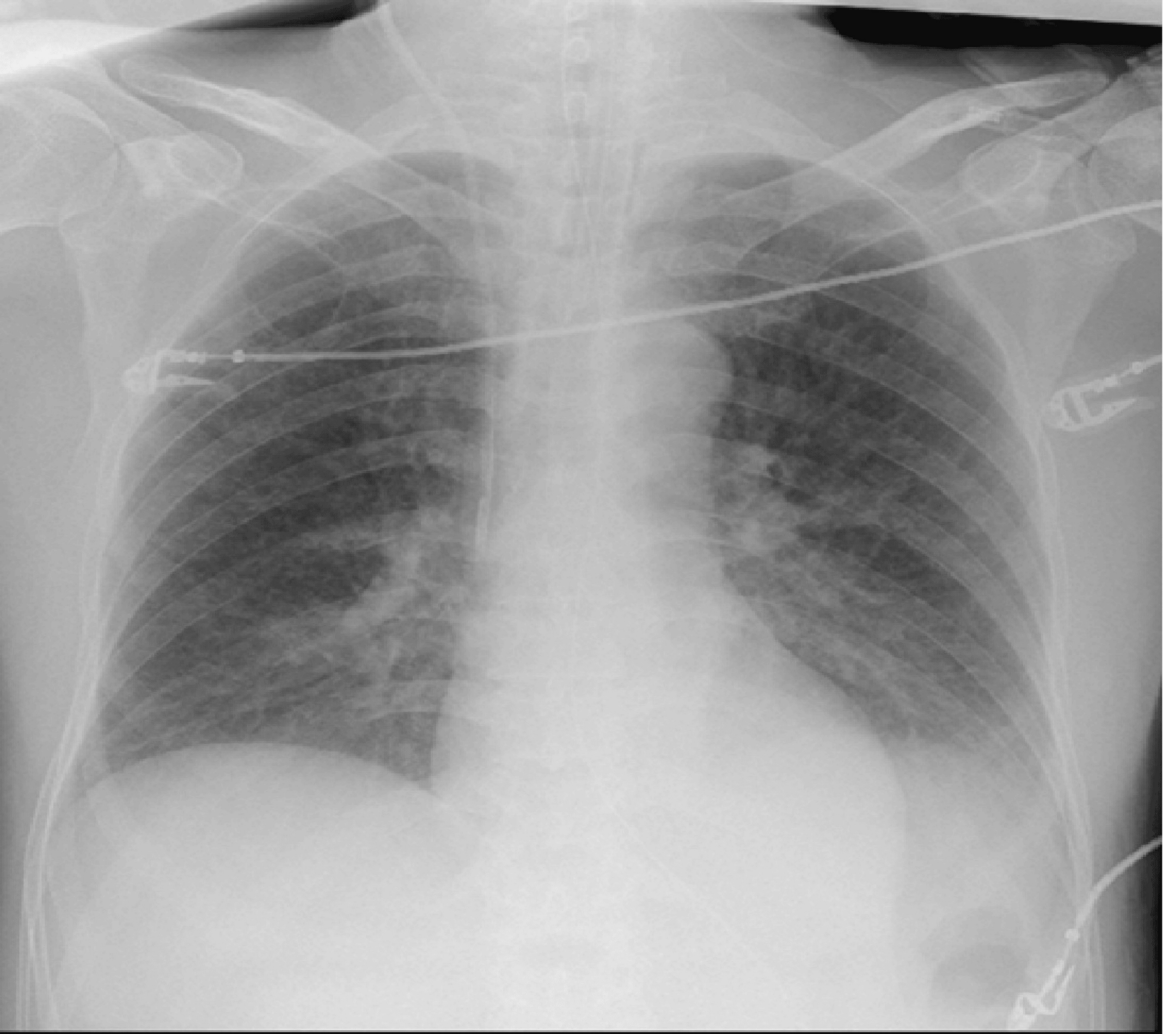
Chest radiograph on the day of discharge from the ICU Chest radiograph showing marked improvement with almost complete resolution of pulmonary infiltrates

A 57-year-old unemployed man with a history of alcohol use disorder, dyslipidemia, and hypertension presented to the emergency department (ED) and reported having generalized malaise with myalgias, which started about six days prior. He also developed abdominal pain, nausea, and vomiting, and noticed a yellowish discoloration of his skin over the past 48 hours. On initial assessment, his vital signs were systolic blood pressure 91 mmHg, heart rate 99 beats per minute, temperature 36.8°C, respiratory rate 15 breaths per minute, and oxygen saturation 98% on room air. Physical examination revealed jaundice and mild tenderness in the right flank without other significant findings. The patient lived with his two sons in unsanitary conditions in a rural environment with frequent contact with rodents. The initial blood gas analysis (BGA) showed a pH of 7.37, pCO2 36 mmHg, pO2 79 mmHg, an oxygen saturation of 97%, bicarbonate 21.8 mEq/L, sodium 131 mEq/L, potassium 3.3 mEq/L, and lactate of 1.2 mmol/l. His admission lab work revealed a hemoglobin of 10.3 g/dL, leucocytes of 18.03x10^9/l, platelets of 26x10^9/L, blood urea of 139 mg/dL, serum creatinine of 4 mg/dL, aspartate aminotransferase of 161 IU/L, alanine aminotransferase of 102 IU/L, gamma-glutamyl transferase of 102 IU/L, total bilirubin of 12 mg/dL, C-reactive protein of 19.83 mg/dL, procalcitonin of 6.3 mg/dL, and albumin of 2.8 g/dL, and coagulation studies were normal (Table 1). Urinalysis revealed mild proteinuria, pyuria, and hematuria. Two sets of blood and urine cultures were collected, and the patient was started on broad-spectrum antibiotics with piperacillin/tazobactam. To rule out an intra-abdominal cause for the clinical picture, an abdominal ultrasound and a CT scan of the abdomen were ordered, both of which were unremarkable. HIV testing and serologies for common viral hepatitis agents (A, B, C, E) were negative. Approximately five hours after ED arrival, the patient developed dyspnea with an increased need for supplemental oxygen. A chest radiograph revealed bilateral infiltrates (Figure 1), prompting consultation with the critical care team. On examination, he exhibited significant respiratory distress (38 breaths per minute) despite a high-concentration non-rebreather oxygen mask. He developed hypotension unresponsive to fluid resuscitation, necessitating norepinephrine. Pre-intubation BGA showed pH 7.13, pCO2 43 mmHg, pO2 39 mmHg, HCO3- 14.3 mEq/L, and lactate 5.6 mmol/L. A bedside cardiac ultrasound revealed a normal systolic function, no apparent chamber dilation, and no evidence of pulmonary embolism. The Sequential Organ Failure Assessment (SOFA) score was 17, indicating severe multiorgan dysfunction. Rapid sequence intubation was performed with direct laryngoscopy, and blood was observed in the airway. Post-intubation endotracheal tube suctioning revealed blood in abundance. Despite positive end-expiratory pressure titration to 10 cmH2O and 100% FiO2, the patient's PaO2/FiO2 ratio was 68. The patient met the criteria for severe ARDS, and protective ventilation was combined with pronation. During the first 24 hours, the patient required frequent tracheal aspiration with copious amounts of blood. The chest reassessment radiograph demonstrated progression of the pulmonary infiltrates (Figure 2), and the complete blood count revealed a drop in the hemoglobin level of 4 g/dl. Two units of red blood cells, one unit of platelets, and tranexamic acid were administered. Although the differential diagnosis for alveolar hemorrhage is broad, given the clinical picture, epidemiological information, and laboratory findings, it was reasonable to suspect leptospirosis SPHS. Due to the high predicted mortality, we opted to send serum and urine samples for polymerase chain reaction (PCR) testing to our national reference laboratory. Serology for leptospirosis and autoantibodies (anti-glomerular basement membrane, antinuclear antibodies, and antineutrophil cytoplasmic antibodies) was also ordered. The patient remained anuric in the first 12 hours of admission, and continuous venovenous hemodiafiltration, without anticoagulation, was initiated. We opted to switch the initial antimicrobial choice to ceftriaxone and maintain it for seven days. The blood cultures, Leptospira serology, and autoantibodies were negative. Due to the clinical severity, a bronchoscopy was deemed unsafe and was not performed. With no confirmed diagnosis and a possibility of autoimmune etiology, as well as anecdotal evidence for the use of corticosteroids in severe leptospirosis, the decision was made to initiate a pulse dose of methylprednisolone (1000mg) for three days and reduce to one milligram per kg, followed by a slow tapering over seven days. The patient kept improving (Figure 3) and was liberated from the ventilator and extubated after 13 days. Although bilirubin levels worsened transiently (peaking at 25 mg/dL), the condition resolved without intervention. The patient was diagnosed with ICU-acquired myopathy. The PCR results arrived on the 12th day after admission, confirming the diagnosis of leptospirosis; both the serum and urine samples were positive. After a lengthy rehabilitation, the patient made a full recovery and was discharged home.

**Table 1:** Timeline of laboratory results and organ support INR: International normalized ratio; pCO2: partial pressure of carbon dioxide; pO2: partial pressure of oxygen; HCO3-: Bicarbonate; FiO2: partial pressure of carbon dioxide; PEEP: partial pressure of carbon dioxide; PaO2/FiO2: ratio of partial pressure of oxygen in arterial blood (PaO2) to the fraction of inspiratory oxygen concentration (FiO2)

Parameter	Unit (reference range)	Emergency Department admission	Before intubation	Day 1	Day 2	Day 3	ICU discharge
Leukocytes	G/L (4.50 – 11.50)	18		17.4	18.1	15.4	7.37
Neutrophils	%	80		83	84	81	51
Platelets	G/L (150 – 450)	26		29	29	32	257
Hemoglobin	g/dL (14-18)	10.3		6.3	8.6	8.7	8.3
Prothrombin time	s (11.7-15.3)	14.1		17.8	13.1	12.4	13.8
INR	-	1.08		1.38	1	0.95	1.06
Activated partial thromboplastin time	s (25 – 34.0)	29.2		35	29.5	26.2	33.6
Urea	mg/dL (19–49)	139		170	106	63	52
Creatinine	mg/dL (0.6–1.3)	4		5.5	3.1	1.8	1
Albumin	g/dL (3.5-4.5)	2.8		3.3			3.6
Aspartate aminotransferase	IU/L (4-43)	161		98	69	46	23
Alanine aminotransferase	IU/L (4-43)	102		70	77	74	50
Alkaline phosphatase	IU/L (25-100)	89		47	54	71	80
Gamma-glutamyl transferase	IU/L (0-73)	102		54.4	56	96	32
Total bilirubin	mg/dL (0.3-1.2)	12		15.6	17.1	16.7	2.8
Direct bilirubin	mg/dL (0-0.3)	9		11.88			2.2
C-reactive protein	mg/dL (<0.50)	19.83		17.44	20.44	11	0.01
Procalcitonin	mg/dL (<0.50)	6.33		50	21		-
pH	7.35-7.45	7.37	7.13	7.18	7.34	7.37	-
PCO2	mmHg (35-45)	36	43	50	42	47	-
PO2	mmHg (80-100)	79	39	107	98	155	-
HCO3-	mEq/L (22-26)	21.8	14.3	19.4	23.7	28.3	-
Lactate	mmol/L (0.5-1.6)	1.2	5.6	1.7	1.5	1	-
FiO2	%	21	60	65	70	50	21
PEEP	cm H2O	-	-	10	9	9	-
PaO2/FiO2			65	165	140	310	-
Urine output	ml/Kg/h			0	0	0	-
Prone position				Yes	Yes	No	-
Renal replacement therapy				Yes	Yes	Yes	-
Driving pressure				-	17	10	-
Norepinephrine				Yes	Yes	Yes	-
Neuromuscular blocking agent				Yes	Yes	Yes	-

## Discussion

Severe leptospirosis is classically described as a biphasic illness, characterized by an initial septicemic phase followed by an immune phase, during which the more severe manifestations occur. With a mean incubation period of 10 days, the septicemic phase, lasting five to seven days, is characterized by high fever and flu-like symptoms. During this phase, leptospires can be detected in both blood and cerebrospinal fluid (CSF). A brief improvement in symptoms may precede the immune phase, where patients can present with jaundice, renal failure, cardiac arrhythmias, meningitis, encephalitis, hepatosplenomegaly, abdominal pain, dyspnoea, haemoptysis, respiratory failure, and shock. In this phase, leptospires can be detected in urine from the second week to several weeks later [8].

One important issue regarding leptospirosis is how to make the diagnosis. Diagnostic methods for leptospirosis can be categorized into direct and indirect detection techniques. The most common diagnostic approach is the indirect method by serology through paired acute and convalescent serum samples, demonstrating seroconversion. The reference standard serological test is the microscopic agglutination test (MAT), but it's difficult to perform and expensive. The IgM enzyme-linked immunosorbent assay (ELISA) is easier to perform but requires confirmation by MAT. Although sensitivity and specificity depend on several factors, MAT has a reported sensitivity of 90% and specificity of >90%, and ELISA has a sensitivity of >90% and a specificity of 88%-95% [1]. However, these methods have several limitations, including only being able to detect antibodies after 7-10 days after the onset of the disease, delayed seroconversions, the possibility of persistence of IgM antibodies that reduces the ability to diagnose acute infection, and cross-reactivity with other pathogens, which can reduce specificity.

Direct methods include culture, which is difficult, slow, and has an extremely low sensitivity (5%-50%) [1], while direct visualization of leptospirosis in blood or urine via dark-field microscopy is unreliable. Molecular techniques to detect leptospiral DNA offer several advantages, including high sensitivity (100%) and specificity (93%) [1]. Using this method on CSF, urine, or blood samples facilitates early diagnosis, however, it is more expensive and may not always be technically feasible. Other direct detection options include staining methods, though these are infrequently used and have low sensitivity [9].

In this case, the patient presented with multiorgan dysfunction and a high probability of death, making a timely diagnosis critical. Given the clinical suspicion of severe leptospirosis in the immune phase, urine samples theoretically had a higher probability of detecting the pathogen. However, the patient was anuric for the first 48 hours. Despite the lower likelihood of detection, a serum sample was also collected and sent for analysis. A second urine sample was later obtained, and both samples tested positive for *Leptospira*. Despite the biphasic nature of the disease, many patients don’t have a clear distinction between these two phases and may present symptoms solely in the immune phase. Given the strong suspicion of an infectious cause, antimicrobial therapy was maintained, switching from the initial choice of piperacillin/tazobactam to ceftriaxone.

There appears to be no difference between penicillin and ceftriaxone for the treatment of leptospirosis, and without a confirmed diagnosis, the broader spectrum of ceftriaxone was a safer choice. A 2012 Cochrane review seems to show conflicting evidence about mortality, and a review of four trials that compared antibiotics with placebo and reported mortality showed no clear benefit of their use in severe illness, but they may reduce the number of days of clinical illness [10]. A dysregulated inflammatory response is the hallmark of sepsis and is common in critically ill patients. While the use of corticosteroids in infection remains controversial, their broad anti-inflammatory properties make them a logical option for managing a hyperinflammatory state. Current evidence suggests a benefit of corticosteroid use in several conditions, such as septic shock and ongoing requirement for vasopressor therapy, severe community-acquired pneumonia, pneumococcal meningitis, etc. However, corticosteroids have notable drawbacks, including interference with the hypothalamic-pituitary-adrenal axis, lack of specificity, and potent immunosuppressive effects. Future treatments may offer more targeted immunomodulatory therapies with fewer immunosuppressive risks. The use of corticosteroids in severe leptospirosis is similarly controversial. A systematic review found some studies suggesting potential benefit using different doses and durations of treatment, although the only randomized controlled trial showed no benefit and potential harm [11]. There is no definitive evidence to support the routine use of corticosteroids in this setting. In this case, the differential diagnosis included the possibility of diffuse alveolar hemorrhage secondary to autoimmune or inflammatory disease, and corticosteroid therapy was initiated, mainly because of this consideration.

This case underscores the importance of a multidisciplinary and timely intervention approach in severe leptospirosis, balancing the use of empiric treatments while awaiting confirmatory testing.

## Conclusions

Leptospirosis presents a diagnostic challenge due to its wide range of clinical manifestations. Fortunately, most cases are benign and self-limiting. However, in its most severe forms, the disease can be life-threatening, with leptospirosis-associated severe pulmonary hemorrhagic syndrome identified as the most lethal complication. Supportive and multidisciplinary care is essential in severe leptospirosis and may include hemodialysis for renal failure and early intubation with protective ventilation for pulmonary hemorrhage with respiratory failure. There is no standard of care for this disease; the use of antimicrobials for mild or severe disease lacks evidence and seems to have no impact on mortality. Further studies are needed to clarify the usefulness of antimicrobials and corticosteroids in severe leptospirosis.
